# Border Health Strategic Initiative: Overview and Introduction to a Community-based Model for Diabetes Prevention and Control^1^

**Published:** 2004-12-15

**Authors:** Stuart J Cohen, Maia Ingram

**Affiliations:** Mel and Enid Zuckerman Arizona College of Public Health; Mel and Enid Zuckerman Arizona College of Public Health, Tuscon, Ariz

## Introduction

This article describes an effort to develop and implement a comprehensive, community-based approach to diabetes prevention and control in selected communities along the U.S.-Mexico border.

The U.S. state of Arizona shares a border with the Mexican state of Sonora. The four Arizona counties on this border are Cochise, Santa Cruz, Pima, and Yuma. In 1996, the University of Arizona and the Arizona Department of Health Services conducted a diabetes survey in the city of Douglas, Cochise County, in conjunction with its U.S. community partners and Mexican counterparts (1). This partnership led to the formation of a diabetes working group in Douglas. In 1998, members of this working group and other stakeholders formed the community advisory board of the newly funded Prevention Research Center (PRC) at the University of Arizona.

In 1999, the PRC community advisory board urged university faculty to focus on studies that prevent or control diabetes. As members also of border communities, community advisory board members felt the personal impact of diabetes among Hispanics. Hispanic Americans are now the largest and fastest growing minority group in the United States, with an estimated growth from 30 million (or 11% of the U.S. population) in 1998 to 97 million (or 25% of the U.S. population) by 2050 (2). In 2000, approximately 2 million of the 30 million Hispanic Americans were diagnosed with diabetes — 1.9 times the rate seen in non-Hispanic whites (2). Among Hispanic Americans aged ≥50 years, 25% to 30% have diagnosed or undiagnosed diabetes (2). Risk factors for diabetes (e.g., family history of diabetes, gestational diabetes, obesity, physical inactivity) are more common among Hispanic whites than non-Hispanic whites (2). Mexican Americans, who make up 64.3% of the total U.S. Hispanic population and live primarily in the south-central and southwestern United States, have the highest rate of diabetes among Hispanic Americans (1). They are twice as likely to have diabetes and have higher rates of diabetic nephropathy, retinopathy, and peripheral vascular disease than non-Hispanic whites (2).

**Figure:**
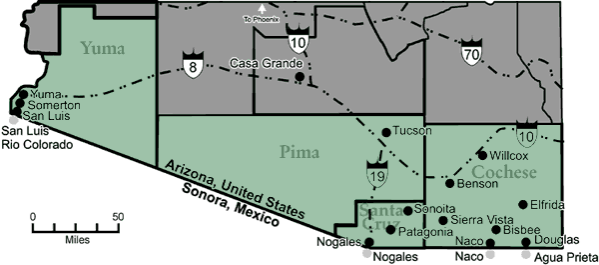
The Arizona–Sonora border

On the U.S.-Mexico border, the impact of diabetes is reaching epidemic proportions. Based on a random household survey of Hispanic populations aged ≥40 years in two Arizona border counties, Pima and Santa Cruz, the prevalence of diabetes was 20%, which is 2–2.5 times higher than non-Hispanic whites (3). In this survey, diabetes was defined as either an affirmative response to the question of whether diabetes had been diagnosed by a physician or having an HbA1c blood test of 7.0% or greater. Between 1995 and 1997, diabetes was the fourth leading cause of death in Mexican communities on the border (4). Furthermore, type 2 diabetes is being diagnosed in increasingly younger individuals, including children and adolescents (5). Reversing these trends would require a comprehensive community-oriented approach focused on diabetes prevention and control (6,7).

The University of Arizona obtained a federal appropriation to develop a comprehensive, community-based approach to diabetes prevention and control. Fortunately, a number of community members and university faculty had a long and successful record of working together. The funding for the initial year of the project was for slightly more than $1 million. We subsequently obtained an additional $1.5 million to continue the project for two more years. The Division of Diabetes Translation at the Centers for Disease Control and Prevention was the project administrative agency.

A major factor in the collaboration was a history of a close working relationship in health promotion efforts between faculty from the Mel and Enid Zuckerman Arizona College of Public Health and Cooperative Extension agents from the College of Agriculture and Life Sciences. The advantage of Cooperative Extension is that each county has a designated agent who resides in that county and knows the key organizations and leaders. In addition, to assure the involvement of community agencies, almost half of the budget went directly to them through nine subcontracts. The project was known as the Border Health Strategic Initiative, or, more usually, *Border Health ¡SI!.*

## Setting

The *Border Health ¡SI!* effort was primarily concentrated in Yuma and Santa Cruz counties. The communities of Somerton and San Luis are located in southern Yuma County, 251 miles southwest of Tucson. Together the communities have 22,588 residents and share the international border with the city of San Luis Rio Colorado, Mexico, which is home to 126,600. Both communities have governmental structures consisting of a mayor, city council, and city manager and interface with a broader board of Yuma County supervisors. Yuma Regional Medical Center is the only hospital serving the entire county, and Sunset Community Health Center is the federally funded community health center serving both Somerton and San Luis. The local county health department is responsible for public health functions throughout the area. Western Arizona Area Health Education Center and the local University of Arizona Cooperative Extension Office both support local health professional education and community health education throughout the region. Other nonprofit organizations provide strong outreach efforts through community health outreach workers. Both Somerton and San Luis are predominantly Hispanic (95.2% and 89.1%, respectively). Poverty is an important issue in both communities, with 26.4% of Somerton residents and 35.6% of San Luis residents reporting incomes below 100% of the federal poverty level. Furthermore, 62.2% of the population in Somerton and 77.5% of the San Luis population report incomes below 200% of the federal poverty level. Unemployment rates are extremely high in both communities; 57.2% of the Somerton population and 61.3% of the San Luis population reported unemployment in the year 2000. Additionally, educational levels are low within the primary care area of each community; 57.3% of Somerton residents and 68.5% of San Luis residents report having less than a high school education.

In Santa Cruz County, our partner community is Nogales. Located 70 miles south of Tucson at the U.S.-Mexico border, Nogales has 20,878 residents. Nogales, Sonora, which is located across the international boundary in Mexico, has a population of 156,900 residents. The city has a governmental structure of a mayor, city council, and city manager. The local government also interfaces with a broader board of supervisors, which provides the governmental framework for Santa Cruz County. The primary health care facilities in the area include Holy Cross Hospital and Mariposa Community Health Center, a federally funded community health center. Mariposa Community Health Center and the local county health department share responsibility for public health functions throughout the area. The Southeastern Arizona Area Health Education Center and the local University of Arizona Cooperative Extension Office collaborate to provide professional health education and community health education in the region. The vast majority (93.6%) of Nogales residents are Hispanic, with 33.9% reporting an income 100% below the federal poverty level and 64.3% reporting an income below 200% of the federal poverty level. Almost one fifth (18.4%) of the Nogales population was unemployed in 2000 and within the broader Nogales primary care area; 47.6% of the population has less than a high school degree.

## Intervention

The *Border Health ¡SI!* approach borrowed heavily from models of community capacity building and community change (8-13). The *Border Health ¡SI! *university-community partnerships were designed to be comprehensive, community-oriented, acceptable to stake holders, effective in fostering and sustaining change, adaptable to other communities, and sustainable after the funding ceases, and the approach included process and outcome assessment.

In efforts to prevent type 2 diabetes, *Border Health ¡SI! *addressed risk factors such as obesity (related to diet and lack of physical activity), family history, and age. Changes in lifestyle, such as improved diet, increased physical activity, and modest weight loss, have been shown to prevent diabetes in individuals with impaired glucose tolerance (14). In addition, because of the growing incidence of diabetes among adolescents, the intervention targeted schools.

Although diabetes prevention was an important focus for the project, we also decided to target patients diagnosed with diabetes, their families, and their health care providers at the community health centers where most patients received their care. Community health outreach workers — or *promotores de salud* — were instrumental to the success of interventions designed to change personal health risk factors such as improper nutrition and inadequate physical activity. *Promotores de salud* have been used extensively in Latin America and in communities of color and have been shown to be effective (15,16).

The *Border Health ¡SI!* model included the following program components: patient self-management, quality of care improvement, patient family prevention and support, community nutrition and exercise, and school health policy (Figure). Participants in each component, as well as other community leaders, formed community-based coalitions called Special Action Groups (SAGs) to identify and implement plans for policy and environmental changes. Each case study in this issue of *Preventing Chronic Disease* pertains to a target population in the model. By clicking on the link in the model, readers will be directed to the case study for that component. The SAG is the "glue" that holds all the components together. *Promotores de salud* were key to all but the provider and school components.

**Figure:** Model for the Border Health Strategic Initiative, Yuma and Santa Cruz counties, Arizona, 2004.

## Outcomes

The community case studies in this issue of *Preventing Chronic Disease* describe and analyze the results of the interventions. Beyond those results, however, the synergy created through the *Border Health ¡SI!* effort had an impact on two important areas. The first was in building the capacity of the communities to work together to create comprehensive, integrated, and sustainable diabetes prevention and control programs. The second was the overwhelming success of this academic-community partnership to integrate both research and community perspectives to address the devastating toll of diabetes.

*Border Health ¡SI!* brought together community agencies and the university with comprehensive funding, broad vision, united mission, and policy focus. The fact that each agency had a specific, funded role and area of expertise in the project resulted in mutual ownership of both the issue and the strategies used to address it. The cooperative extensions in both counties played a vital role in supporting the efforts of the other agencies by providing leadership to the partnership, offering resources for promotional items, helping to coordinate events, and acting as a liaison between all the partners. The *promotores de salud *responsible for carrying out the project components extended themselves much further — for example, by participating in food demonstrations in one community and by collecting signatures for a petition to create a community park in another.

The collaborative relationships created through *Border Health ¡SI! *also contributed to the sustainability of the components. Using the experience and results from the interventions, both communities pursued and obtained additional funding to continue and expand their efforts. *Border Health ¡SI! *partners and other community leaders continue to meet regularly to address diabetes through their SAGs and in other partner efforts.

*Border Health ¡SI!* was based on a history of collaborative efforts between the University of Arizona and community partners. This project, however, expanded the number and substance of involvement of those in the academic institution within a community setting. The curriculum for the family component, for example, was developed collaboratively between academics and *promotoras* who had never worked together in the past. The community partners were satisfied by the relationship because the entire premise of the project was based on the needs of the community and they were equal partners in the process. Both partners benefited from the focus on measuring outcomes — in learning to appreciate the role of research in a community intervention and in securing funding for future efforts. The partnership was also a great benefit to academic individuals: while they provided expertise in creating community interventions, they learned from community members with expertise in implementing them.

## Conclusions

Multiple and diverse efforts are required to have a sustainable impact on the prevention and control of diabetes in the community context. In addition to implementing and sustaining community-wide programs, the community must work to attain policy and environmental changes that support behavior changes. Grassroots leadership provided by community health workers, combined with community coalition efforts, can bring together diverse sectors of the community to generate positive outcomes. Academic-community partnerships contribute to technical capacity building at the community level and heighten sensitivity among academics to the complexity of issues faced by the community and the talent and resolve of community members to address them.

## Acknowledgments

We thank our community partners for their dedication to improving their communities: University of Arizona Cooperative Extension Offices in Yuma and Santa Cruz counties, Mariposa Community Health Center, Southeast Regional Health Education Center, Carondolet Medical Network, Sunset Community Health Center, Puentes de Amistad, Campesinos Sin Fronteras, and Western Arizona Health Education Center. We also thank the *promotores de salud* in Yuma and Santa Cruz counties without whose help this project would not have been possible. Funding for this project is provided by the Division of Diabetes Translation, Centers for Disease Control and Prevention.
